# Serotonin inhibits low-threshold spike interneurons in the striatum

**DOI:** 10.1113/jphysiol.2011.219469

**Published:** 2012-04-10

**Authors:** Sarah Cains, Craig P Blomeley, Enrico Bracci

**Affiliations:** Faculty of Life Sciences, University of Manchester, AV Hill BuildingOxford Road, Manchester M13 9PT, UK

## Abstract

Low-threshold spike interneurons (LTSIs) are important elements of the striatal architecture and the only known source of nitric oxide in this nucleus, but their rarity has so far prevented systematic studies. Here, we used transgenic mice in which green fluorescent protein is expressed under control of the neuropeptide Y (NPY) promoter and striatal NPY-expressing LTSIs can be easily identified, to investigate the effects of serotonin on these neurons. In sharp contrast with its excitatory action on other striatal interneurons, serotonin (30 μm) strongly inhibited LTSIs, reducing or abolishing their spontaneous firing activity and causing membrane hyperpolarisations. These hyperpolarisations persisted in the presence of tetrodotoxin, were mimicked by 5-HT_2C_ receptor agonists and reversed by 5-HT_2C_ antagonists. Voltage-clamp slow-ramp experiments showed that serotonin caused a strong increase in an outward current activated by depolarisations that was blocked by the specific M current blocker XE 991. In current-clamp experiments, XE 991 *per se* caused membrane depolarisations in LTSIs and subsequent application of serotonin (in the presence of XE 991) failed to affect these neurons. We concluded that serotonin strongly inhibits striatal LTSIs acting through postsynaptic 5-HT_2C_ receptors and increasing an M type current.

**Key points**

- The striatum is the largest nucleus of the basal ganglia, a brain structure crucially involved in motor control. Recent results show that nitric oxide plays an important role in striatal pathophysiology.
- The activity of the striatum is modulated by extrinsic neurotransmitters such as serotonin, produced by specialised neurons located in the brainstem.
- This modulation is exerted through control of striatal interneurons. However, nitric oxide-producing interneurons (NOS interneurons) have been difficult to investigate due to their rarity.
- Using transgenic mice in which NOS interneurons express green fluorescent protein, we found that NOS interneurons are strongly inhibited by serotonin.
- This inhibition is mediated by a specific class of serotonin receptors (5-HT_2C_) causing an increase in a specific potassium conductance (KCNQ).
- These results cast light on the role of serotonin in the striatum, revealing that it tightly controls the activity of the only neuronal type that releases nitric oxide.

## Introduction

The striatum is the main input nucleus of the basal ganglia and plays a critical role in motor control, reward-mediated learning and action selection (Redgrave *et al.* 1999; Graybiel, 2005). This area receives strong glutamatergic inputs from the cortex and thalamus; this input is processed by complex local circuits that determine the striatal output, which in turn shapes the activity of the other basal ganglia nuclei (Bolam *et al.* 2000). The activity of striatal circuits is controlled by extrinsic neuromodulators. The crucial role of dopamine (released in the striatum by the axons of midbrain neurons located in the substantia nigra pars compacta and ventral tegmental area) has been widely recognised since the 1960s (Gerfen & Surmeier, 2011). On the other hand, the importance of the dense serotonergic projections to the striatum from the raphe nuclei (Lavoie & Parent, 1990) has been recognised more recently. Serotonin regulates a variety of physiological processes including arousal, cognition and mood (Monti, 2011); furthermore, a subpopulation of serotonergic neurons in the raphe nuclei display phasic activation during specific types of motor behaviour (Fornal *et al.* 1996). In the striatum, serotonin was recently found to induce a form of long-term depression at corticostriatal synapses (Mathur *et al.* 2011). Furthermore, we have shown that serotonin strongly excites two classes of striatal interneurons (Blomeley & Bracci, 2005, 2009). There is also evidence for a role of serotonin in Parkinson's disease; chronic dopamine depletion has been reported to cause serotonin depletion, which co-exists with an increase in serotonergic axonal arborisations in the striatum (Bedard *et al.* 2011).

While projection cells account for up to 95% of the neurons in the striatum, several classes of interneurons play an essential role in striatal information processing (Tepper & Bolam, 2004) and have been recognised in the past decade as crucial targets for extrinsic neuromodulators such as dopamine and serotonin (Pisani *et al.* 2000; Bracci *et al.* 2002; Blomeley & Bracci, 2005, 2009). Striatal interneurons are comprised of cholinergic cells and different types of GABAergic neurons (Tepper *et al.* 2010), including fast spiking interneurons and low-threshold spike interneurons (LTSIs). The LTSIs also produce somatostatin and neuropeptide Y (NPY), and are the only striatal neurons that express nitric oxide synthase. LTSIs have been comparatively less studied than other interneurons due to their numerical paucity; nevertheless, their unique biochemical machinery (Kawaguchi, 1993; Partridge *et al.* 2009) and the large size of their dendritic and axonal arborisation (Kubota & Kawaguchi, 2000) suggest that they play an essential role in the striatal circuitry. LTSIs express neuropeptide Y (NPY) (Kawaguchi, 1993); the recent introduction of a BAC mouse strain, in which green fluorescent protein (GFP) is expressed under control of the neuropeptide Y (NPY) promoter (Partridge *et al.* 2009), has made systematic electrophysiological investigations of these cells possible. We used this mouse strain to investigate how serotonin affects the LTSIs. We describe that, in sharp contrast with the excitatory effects of serotonin on cholinergic and fast spiking interneurons, this neurotransmitter strongly inhibits LTSIs.

## Methods

BAC mice, in which the neuropeptide Y (NPY) promoter was attached to a humanized *Renilla* GFP (BAC-*npy*; Stock 006417, Jackson Laboratory, Bar Harbor, ME, USA) were bred in-house at the Biological Services Facility, University of Manchester. All animals used were heterozygous, resulting from heterozygous BAC-*npy* trangenic and wild-type crossings. NPY-expressing neurons in the striatum coincide with LTSIs (Partridge *et al.* 2009). LTSIs in these mice were identified through epifluorescent excitation of the slice with a mercury lamp (Olympus U-RFL-T) coupled with standard GFP filters. In rats, medium-sized cells were targeted and subsequently identified as LTSI if they displayed the distinctive membrane properties of these cells (described in the Results).

For all experiments, rats and mice (both sexes) were killed by cervical dislocation in accordance with the UK Animals (Scientific Procedures) Act 1986 and the European Communities Council Directive (86/609/EEC). Sagittal brain slices (250 μm thick) were cut using a vibroslicer, and maintained at 25°C in oxygenated (Carbogen, 95% O_2_–5% CO_2_, artificial cerebro-spinal fluid (ACSF); composition (in mm): 126 NaCl, 2.5 KCl, 1.3 MgCl_2_, 1.2 NaH_2_PO_4_, 2.4 CaCl_2_, 10 glucose, 18 NaHCO_3_). For recordings, slices were submerged, superfused (2–3 ml min^−1^) at 25°C and visualized with infrared/differential interference contrast microscopy after identification of an LTSI using epifluorescence. Drugs were prepared in stock solutions and bath applied at known concentrations via a gravity system. With this system, ligands reached the slice approximately 2 min after the start of the application.

Conventional current-clamp recordings were performed in bridge mode using an Axoclamp-2B amplifier or a NPI BA-1S bridge amplifier. Voltage-clamp recordings were performed using the AxoClamp-2B in continuous single-electrode mode, with partially compensated series resistance (to reduce the risk of oscillations). Whole-cell recordings were obtained with patch pipettes (3–5 MΩ) filled with a solution containing (mm): 125 potassium gluconate, 10 NaCl, 1 CaCl_2_, 2 MgCl_2_, 1 BAPTA, 19 Hepes, 0.4 Mg-GTP and 4 Mg-ATP, and adjusted to pH 7.3 with KOH. Resting membrane potential was measured under current-clamp conditions in the absence of any injected current.

In voltage-clamp experiments, slow voltage ramps (10 mV s^−1^) were applied; the pre-ramp holding potential was –70 mV and the ramp started from –110 mV. In order to avoid transient currents at the beginning of the ramp, these ramps were preceded by a negative ramp (−10 mV s^−1^) from –70 mV to –110 mV.

The junction potential between the intra-pipette solution and the ACSF was measured (Neher, 1992) and its value (10 mV) was subtracted from all voltage measurements

Cell-attached recordings were obtained with electrodes similar to those used for whole-cell recordings, filled with ACSF. A membrane seal (>1 GΩ) was obtained as in whole-cell recordings, but the membrane was not subsequently ruptured. Under these conditions, spontaneous spikes were readily identified as rapid, biphasic deflections of the recorded potential.

Experimental values are expressed as mean ± standard deviation and statistical comparisons were carried out using the non-directional, Student's unpaired *t* test unless otherwise specified. The threshold level of significance for all analyses was *P* < 0.05. For each individual LTSI, the effect of a pharmacological treatment on firing frequency was assessed by comparing statistically the inter-spike intervals (ISIs) for the different pharmacological conditions. In each condition, at least 50 consecutive ISIs were used for statistical analysis.

Drugs were obtained from Tocris Bioscience UK, apart from 5-HT hydrochloride, which was obtained from Sigma-Aldrich UK.

## Results

### Electrophysiological identification of striatal low-threshold spiking interneurons

We used 22 Sprague–Dawley rats and 55 BAC-*npy* mice for these experiments. Rats were used at postnatal ages ranging from 14 to 29 days (average 21 ± 4 days) and BAC mice were used at postnatal ages ranging from 14 to 34 days (average 22 ± 6 days). We recorded from 109 striatal LTSIs from BAC mice (54 with whole-cell technique and 45 with cell-attached technique); and 22 LTSI from rats (with whole-cell technique). In mice, recordings were obtained from LTSIs located in the central part of the dorsal striatum in sagittal section 1.50 to 2.10 mm lateral to the midline, in an area defined by the following stereotaxic coordinates: 2.5 to 3.5 mm dorsal to the interaural line and 4 to 5 mm rostral to the interaural line. In rats, recordings were obtained from LTSIs located approximately 3.2 to 4.7 mm dorsal to the interaural line and 9.1 to 10.2 mm rostral to the interaural line, in sagittal sections 2.4 to 3.9 mm lateral to the midline.

**Figure 1:**
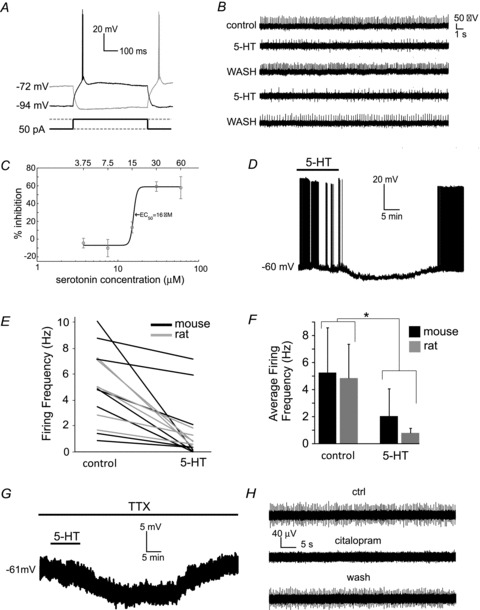
Serotonin hyperpolarises striatal LTS interneurons *A*, typical responses of an LTSI to positive and negative current injections which produce low-threshold calcium spikes during or after the pulse, respectively. *B*, cell-attached recording from an LTSI showing reversible depressing effects of serotonin (5-HT) on spontaneous firing; reapplication of serotonin after washout elicited similar effects as the first application. *C*, dose–response curve for the inhibitory effects of serotonin on spontaneous firing frequency of LTSIs. Results were obtained with cell-attached recordings. For each neuron, the effects of each concentration of serotonin were expressed as percentage of the firing frequency observed in the absence of serotonin. Percentage of inhibition was then defined as (100 – firing frequency in serotonin expressed as percentage of control). A sigmoidal curve was fitted to the data using a Matlab routine. This fit yielded a half-maximal response dose of 16 μm. Each concentration of serotonin was tested in at least 4 LTSIs. *D*, serotonin (30 μm) reversibly hyperpolarised a spontaneously active LTSI and fully blocked action potential generation. *E*, effects of serotonin on spontaneous firing frequency in individual LTSIs from rats or BAC NPY-GFP transgenic mice.*F*, average effects of serotonin on the spontaneous firing frequency of LTSIs from Sprague–Dawley rats or BAC NPY-GFP mice. Asterisk denotes statistical significance (*P < 0.05; assessed with Mann–Whitney *U* test; *n* = 8 for mice and 6 for rats). *G*, in the presence of TTX, serotonin reversibly hyperpolarises LTSIs; a typical example is shown. *H*, cell-attached recording from an LTSI in which the serotonin reuptake blocker citalopram reversibly abolished spontaneous firing.

In BAC mice, LTSIs were initially identified through their epifluorescence using a standard GFP filter set. In both BAC mice and rats, LTSI identification was established based on the presence of their distinctive electrophysiological features including: (i) very high input resistance (>500 MΩ); (ii) relatively depolarised resting membrane potential (more positive than –70 mV); and (iii) ability to generate low-threshold slow spikes (Kawaguchi, 1993; Koos & Tepper, 1999; Partridge *et al.* 2009). Examples of these properties are shown in Fig. 1*A*. Of the cells patched in the absence of the sodium channel blocker tetrodotoxin (TTX), 14/15 from BAC mice and 16/18 LTSIs from rats were spontaneously active. In these cells, the average spontaneous firing frequency was 5.2 ± 3.3 Hz in BAC mice LTSIs and 4.8 ± 2.5 Hz in rat LTSIs. In the LTSIs that were not spontaneously active, the average resting membrane potential (in the absence of any injected current) was −60.2 ± 7.2 mV in BAC mice and −61.5 ± 5.1 mV in rats. The average input resistance was 710 ± 180 MΩ in rat LTSIs and 799 ± 321 MΩ in BAC mice.

### Effects of serotonin on LTSIs and receptors involved

We investigated the effects of serotonin on the excitability of LTSIs. As most LTSIs are spontaneously active, it was possible to monitor their firing activity using cell-attached recordings. In initial experiments, we applied 30 μm serotonin, as in previous studies in striatal cholinergic interneurons and fast spiking interneurons (Blomeley & Bracci, 2005, 2009). In each of 16 cell-attached experiments (in BAC mice), this dose of serotonin caused a reversible decrease in LTSI firing frequency (significant increase in ISI; *P* < 0.001). Similar effects on firing were observed again when serotonin was re-applied after washout (*n* = 4), as shown in the example of Fig. 1*B*. The time from the start of serotonin application to its maximal effects on firing frequency was on average 11 ± 2 min.

In another series of experiments we used cell-attached recordings to test the effects of different concentrations of serotonin. Serotonin reduced spontaneous firing in LTSIs in a dose-dependent manner; a dose–response curve revealed that on average half-maximal effects were observed at ∼16 μm (Fig. 1*C*). Each concentration in Fig. 1*C* was tested on at least four spontaneously active LTSIs (from BAC mice). Based on these results, we decided to use a concentration of 30 μm in the rest of the experiments.

In order to gain further insight into the effects of serotonin, we tested eight LTSIs from BAC mice and six from rat using whole-cell recordings. All these cells were spontaneously active in control solution. Serotonin significantly reduced firing in all LTSIs tested (*P* < 0.001 for the ISI), with a complete and reversible cessation of firing observed in 4/8 LTSIs in BAC mice and 4/6 in rat. A representative example of these experiments is shown in Fig. 1*D*. In whole-cell experiments, the time from the start of serotonin application to its maximal effects was 16.6 ± 5.0 min in BAC mice and 16.1 ± 4.4 min in rats. The effects of serotonin on BAC mice and rat LTSI firing frequency are summarised in the plots of Fig. 1*E* and *F*.

In another series of experiments, we applied serotonin in the presence of TTX (1 μm) in order to establish whether serotonin caused a direct effect on LTSIs. The resting membrane potential of LTSIs in BAC mice (*n* = 5) and rats (*n* = 4) in the presence of TTX was 59.3 ± 4.9 mV and −64.8 ± 4.4 mV, respectively. Serotonin induced hyperpolarising effects in the presence of TTX in 4/5 cases (BAC mice) and 4/4 cases (rats), as shown in the example of Fig. 1*G*. On average, serotonin-induced hyperpolarisations were 6.5 ± 3.3 mV in BAC mice and 8.3 ± 2.2 mV in rats. These hyperpolarisations reached maximal value 16.0 ± 2.6 min after the onset of serotonin application in BAC mice and 22.3 ± 2.9 min in rats. We concluded that the effects of serotonin on LTSIs were direct and did not depend on action potential-mediated synaptic transmission. These results also showed that the effects of serotonin are similar in BAC mice and rat LTSIs. Therefore, we continued our investigation using BAC mice only, where LTSIs could be identified more easily.

In order to establish whether endogenous serotonin could produce similar effects on LTSIs as exogenously applied serotonin, we applied the serotonin reuptake blocker citalopram (1 μm). In 6/7 cell-attached experiments, citalopram reduced or abolished firing (*P* < 0.05 for the ISI), as shown in the example of Fig. 1*H*. In the remaining one case, citalopram did not significantly affect LTSI firing frequency. Thus, increasing endogenous serotonin by blocking its reuptake inhibits LTSI firing in brain slices.

**Figure 2:**
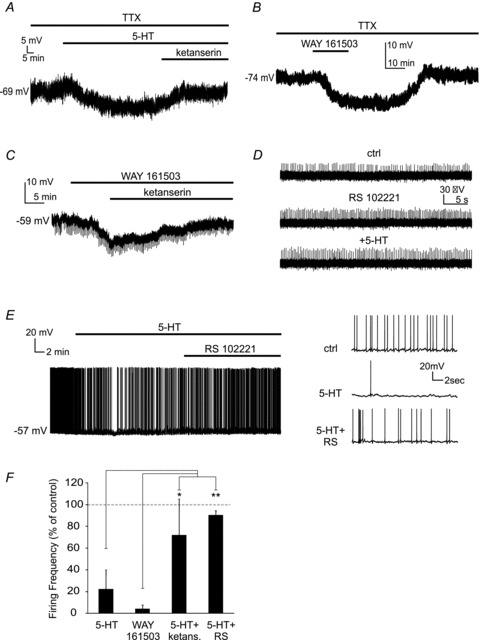
The effects of serotonin on LTSIs are mediated by 5-HT_2C_ receptors *A*, in a representative experiment, serotonin (30 μm) hyperpolarised an LTSI (recorded without any current injection) in the presence of TTX (1 μm). Subsequent addition of ketanserin (10 μm) caused the LTSI to depolarise to control level. *B*, another LTSI (recorded without current injection), with a resting membrane potential of –74 mV, was reversibly hyperpolarised by the 5-HT_2C_ receptor agonist WAY 161503 (10 μm) in the presence of TTX. *C*, in a different LTSI, in the presence of TTX, the hyperpolarising effects of WAY 161503 (10 μm) were reversed by subsequent application of ketanserin (10 μm; still in the presence of WAY 161503). *D*, in a cell-attached experiment, the 5-HT_2C_ receptor antagonist RS 102221 (1 μm) increased spontaneous firing frequency in a LTSI; in the presence of RS 102221, serotonin failed to affect the LTSI firing activity. *E*, in this experiment, an LTSI (recorded without current injection) displayed spontaneous firing activity in control solution. Serotonin induced a hyperpolarisation accompanied by a reduction in spontaneous firing frequency. Subsequent application of the 5-HT_2C_ receptor antagonist RS 102221 (1 μm) repolarised the LTSI and increased spontaneous firing activity. *F*, bar chart showing the average changes in firing frequency (with respect to control solution) caused by combinations of serotonin receptor ligands. Only LTSIs that were spontaneously active in control solution were included in this analysis. Asterisks denote statistical significance (*P < 0.05; **P < 0.001, assessed with Mann–Whitney *U* test; *n* = 14 for serotonin; *n* = 6 for WAY 161503; *n* = 5 for 5-HT + ketanserin; *n* = 5 for 5-HT + RS 102221). Firing frequency in the presence of either serotonin or WAY 161503 was significantly (*P* < 0.001) lower than in control.

In other striatal interneurons, the effects of serotonin are mediated by 5-HT_2_ receptors (Blomeley & Bracci, 2005, 2009). As an initial test for the involvement of these receptors, we applied (in the presence of TTX) the 5-HT_2_ receptor antagonist ketanserin (10 μm) after serotonin application. In 5/5 LTSIs in which serotonin had caused hyperpolarisations (9.3 ± 2.1 mV) ketanserin (applied in the presence of serotonin) fully reversed these effects, causing the membrane potential to return to the level observed in control solution as shown in the example of Fig. 2*A*.

These data indicated that serotonin hyperpolarised LTSIs by acting through 5-HT_2_ receptors. In the striatum, 5-HT_2A_ and 5-HT_2C_ receptor subtypes are abundantly expressed, with 5-HT2_A_ receptors mainly being expressed on the medium spiny projection neurons. We therefore investigated whether the effects of serotonin on LTSIs were mediated by 5-HT_2C_ receptors, using the subtype-specific 5-HT_2C_ agonist WAY 161503. In these experiments, WAY 161503 (1 μm) was applied in the presence of TTX. WAY 161503 produced a reversible hyperpolarising effect (12.4 ± 5.4 mV) similar to that of serotonin in 6/7 LTSIs, as shown in the example of Fig. 2*B*. In one case, the application of WAY 161503 to the LTSI had no effect. In six LTSIs hyperpolarised by WAY 161503, ketanserin was subsequently applied (still in the presence of WAY 161503); in all cases, the effects of WAY 161503 were completely reversed, as shown in the example of Fig. 2*C*. Conversely, application of the 5-HT_2a_ agonist TCB-2 (2 μm) failed to produce measurable effects on the membrane potential or the firing frequency of 5/5 LTSIs tested (not shown).

To further investigate the role of 5-HT_2C_ receptors, we then used the potent and selective 5-HT_2C_ receptor antagonist RS 102221 (1 μm). In 6/6 cell-attached experiments, RS 102221 increased LTSI firing frequency, producing a significant (*P* < 0.001 for the ISI) decrease in the ISI (on average 62.8 ± 17.4% of control in the presence of RS 102221). This suggests that 5-HT_2C_ receptors were tonically activated by basal levels of serotonin in the slices. Furthermore, in the presence of RS 102221 serotonin failed to affect firing frequency in 4/4 LTSIs. An example of these experiments is shown in Fig. 2*D*.

Furthermore, the ability of RS 102221 to reverse serotonin effects was tested in five spontaneously active LTSIs (recorded in whole-cell configuration). In these LTSIs, application of serotonin significantly (*P* < 0.001 for the ISI) reduced the firing frequency to 6.5 ± 6.2% of control; subsequent application of RS 102221, still in the presence of serotonin, significantly (*P* < 0.001 for the ISI) increased firing frequency in 4/5 cases (to 72.5 ± 40.7% of control) as shown in the example of Fig. 2*E*.

The effects of the different combinations of serotonin receptor ligands on the firing frequency of the LTSIs that were spontaneously active in control solution are summarised in Fig. 2*F*. Overall, these results show that the inhibitory effects of serotonin on LTSIs were entirely mediated by 5-HT_2C_ receptors.

### Conductances modulated by serotonin in LTSIs

**Figure 3:**
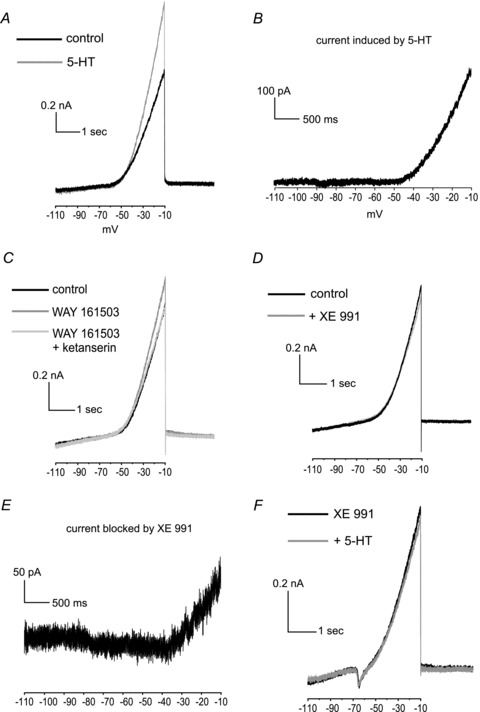
The effects of serotonin on LTSIs are mediated by a reduction in an M type outward current *A*, serotonin increases a voltage-dependent outward current in LTSIs. An example of the voltage-clamp experiments carried out in the presence of TTX. Voltage ramps (from –110 mV to –10 mV, 10 mV s^−1^) were applied to an LTSI, before and after serotonin application. Membrane currents are plotted *vs.* voltage in control solution (black) and in the presence of serotonin (grey). *B*, voltage dependence of the serotonin-induced current in the same LTSI. The steady-state current induced by serotonin was calculated for each voltage by subtracting the membrane current measured in the presence of serotonin from that measured in control solution, before serotonin application. The zero level is indicated by the grey dashed line. *C*, the 5-HT_2C_ receptor agonist WAY 161503 increased a similar voltage-dependent outward current in an LTSI. Subsequent application of the 5-HT_2_ receptor antagonist ketanserin reversed the effect of WAY 161503. *D*, in a representative experiment, XE 991 *per se* reduced a voltage-dependent outward current in LTSIs similar to the one induced by serotonin. Membrane currents are plotted *vs.* voltage in control solution (black) and in the presence of XE 991 (grey). *E*, voltage dependence of the current blocked by XE 991 in the same LTSI. The steady-state current blocked by XE 991 was calculated for each voltage by subtracting the membrane current measured in control solution from that measured after application of XE 991. *F*, in the presence of M current blocker XE 991 (20 μm), serotonin failed to induce an outward current. The zero level is indicated by the grey dashed line.

The observation that the effects of serotonin on LTSIs persisted in the presence of TTX suggested that its action did not involve the voltage-activated sodium currents sensitive to this drug. We investigated the action of serotonin on other ionic currents by carrying out slow voltage-clamp experiments in LTSIs (*n* = 7) in the presence of TTX (see Methods for details). Briefly, a series of voltage ramps (4 s long) were applied in control solution and in the presence of 5-HT (after >20 min from the onset of the application). These ramps covered levels between −110 and –10 mV. In these experiments, serotonin induced an outwardly rectifying current that activated around –50 mV in LTSIs, as shown in the example of Fig. 3*A* and *B*. Similar currents were also induced in 6/7 LTSIs by WAY 161503. In the presence of serotonin or WAY 161503, the LTSI current–voltage relationship had an average slope, in the region between −30 and –10 mV, of 37 ± 7 pA mV^−1^, significantly (*P* < 0.05 with Mann–Whitney *U* test; *n* = 7) larger than that observed in control solution (28 ± 6 pA mV^−1^). The effects of WAY 161503 on the current slope were fully blocked by subsequent addition of ketanserin, applied still in the presence of WAY 161503 (*n* = 6; Fig. 3*C*).

Voltage-dependent potassium channels of the KCNQ family (previously known as M-channels) are typical targets for neuromodulators. The currents induced by serotonin or WAY 161503 in LTSIs activated at potentials slightly more depolarised than typical neuronal KCNQ currents (Brown & Passmore, 2009). However, LTSIs have long dendritic and axonal processes (Kubota & Kawaguchi, 2000); in voltage-clamp experiments, the nominal (somatic) voltage can differ substantially from the actual potential of distal dendrites (Williams & Mitchell, 2008). Thus, we hypothesised that the current induced by serotonin could be through KCNQ channels expressed in dendritic or axonal locations not adequately clamped in voltage-clamp experiments. To test this hypothesis, the specific KCNQ channel blocker XE 991 (20 μm) was applied in the presence of TTX. XE 991 *per se* inhibited an outwardly rectifying current similar to that induced by serotonin (Fig. 3*D* and *E*). Furthermore, in the presence of XE 991, subsequent application of serotonin failed to affect significantly the ramp currents in 5/5 LTSIs (Fig. 3*F*). These data indicated that the effects of serotonin were exerted through the induction of an XE 991-sensitive KCNQ current.

**Figure 4:**
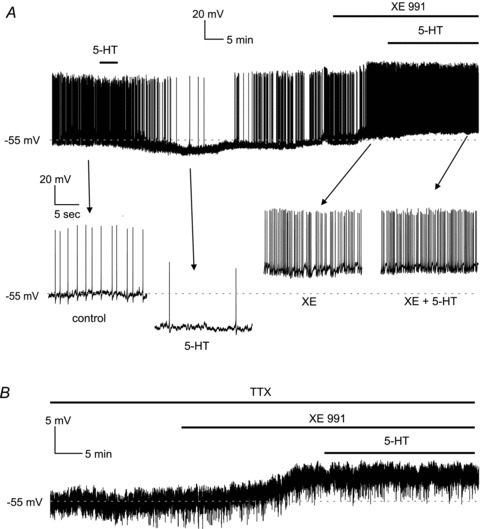
The inhibitory effects of serotonin on LTSIs are blocked by XE 991 *A*, in this LTSI, application of serotonin caused a reversible hyperpolarisation that abolished spontaneous firing activity. After washout of serotonin, application of the M current blocker XE 991 (20 μm) caused a depolarisation accompanied by an increase in spontaneous action potential frequency. In the continuous presence of XE 991, serotonin was reapplied but failed to affect the LTSI membrane potential or its firing frequency. Expanded traces show the changes in spontaneous firing activity in response to serotonin alone and in the presence of XE 991. *B*, in another experiment, in the presence of TTX, application of XE 991 depolarised an LTSI by 4 mV. Subsequent application of serotonin in the presence of XE 991 had no effects on the membrane potential of the LTSI.

Additional experiments using XE 991 (*n* = 4) were carried out in current-clamp conditions. LTSIs were briefly exposed to serotonin, which caused membrane hyperpolarisation (10.1 ± 1.9 mV) and reduced firing frequency (by 62.9 ± 11.7%) similar to previous experiments. Following serotonin washout and membrane repolarisation, XE 991 was applied to the slice. This caused a depolarisation (6.2 ± 1.9 mV) in all LTSIs. Serotonin was then re-applied in the presence of XE 991, but had no further effects on LTSI membrane potential or firing frequency. A representative example of these experiments is shown in Fig. 4*A*. We also performed current-clamp experiments in the presence of TTX (*n* = 4). In 4/4 of these, LTSIs, XE 991 caused a membrane depolarisation (5.5 ± 2.2 mV) in the presence of TTX. Subsequent application of 5-HT had no effect on the membrane potential (Fig. 4*B*).These observations completed the demonstration that serotonin effects on LTSIs were mediated by an enhancement in XE 991-sensitive currents mediated by KCNQ channels.

### Wortmannin prevents the effects of serotonin

**Figure 5:**
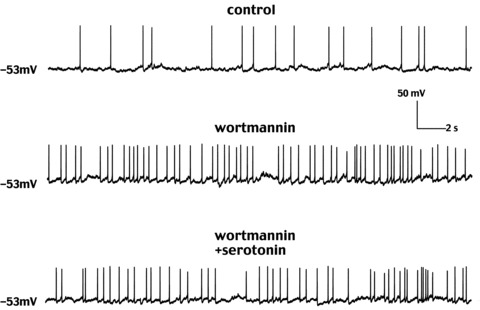
Wortmannin prevents the inhibitory effects of serotonin In this representative experiment, an LTSI's spontaneous firing rate increased significantly (*P* < 0.001 for the ISI) after wortmannin application (10 μm). In the presence of wortmannin, application of serotonin failed to affect the LTSI firing activity or membrane potential.

In other cells, KCNQ channels require membrane phosphatidylinositol-4,5-bisphosphate (PIP_2_) to open (Brown *et al.* 2007). Wortmannin blocks the synthesis of PIP_2_ and interferes with the effects of muscarinic receptor activation (Brown *et al.* 2007). Therefore, we investigated if the effects of serotonin on LTSIs were affected by the presence of this inhibitor. Wortmannin (10 μm) *per se* had excitatory effects in 5/6 LTSIs; of these, three cells were spontaneously active and wortmannin caused a significant (*P* < 0.01 for the ISI) increase in the frequency of spontaneous firing (on average by 59 ± 19%). In the other two cells, that were not spontaneously active, wortmannin caused membrane depolarisations (5.5–7.2 mV). In all cases (6/6), in the presence of wortmannin, subsequent application of serotonin failed to elicit any significant effects on membrane potential or firing frequency. This is illustrated by the representative example of Fig. 5. We concluded that the inhibitory action of serotonin depended on the ability of LTSIs to synthesise PIP_2_.

## Discussion

Our experiments show that LTSIs are strongly inhibited by serotonin through activation of 5-HT_2C_ receptors and that these effects are generated through an increase in an XE 991-sensitive current mediated by KCNQ channels.

The effects of serotonin were rather slow in onset, with typical delays from the start of drug application of 10–20 min in whole-cell recordings. This was not due to a slow perfusion of the slice, as other pharmacological treatments acted within 2–3 min. Whole-cell recording-induced dialysis was partially responsible for the slow time course, but even with non-invasive cell-attached recordings, the effects only appeared after ∼11 min. Similar time courses were previously observed for the effects of serotonin on cholinergic interneurons and fast spiking interneurons (Blomeley & Bracci, 2005, 2009). These observations, together with the recent finding that exposure to serotonin causes long-lasting depression of corticostriatal inputs (Mathur *et al.* 2011), suggest that serotonin does not convey fast (phasic) signals to the striatum, but rather affects the local circuits in a slow (tonic) fashion. This is a major difference with the action of dopamine that, in addition to tonic signals, conveys rapid information about salient and rewarding signals (Schultz, 2007). Endogenous serotonin present in brain slices exerted a tonic action on LTSIs, as firing frequency was increased by a 5-HT_2C_ receptor antagonist. Furthermore, increasing endogenous serotonin concentration in the extracellular space by blocking its reuptake with citalopram strongly inhibited LTSI firing.

The outward currents induced by serotonin in LTSIs during slow voltage ramps activated at somatic potentials slightly more positive than previously reported (Brown & Passmore, 2009). This discrepancy is likely to result from insufficient space clamp of the dendrites and/or axonal process of LTSIs, combined with possible preferential location of KCNQ channels in distal processes. A somatic current injection setting the local membrane potential at about −50 mV will cause a significantly smaller depolarisation in a distal compartment. This phenomenon has been described by Williams and Mitchell (2008) in pyramidal neurons; in these cells, during voltage-clamp experiments carried out with somatic patch electrodes, differences of up to 40 mV were measured between the somatic potential and the real potential of distal dendrites. In the present case, this notion is supported by the fact that serotonin did not elicit any effect in the presence of XE 991, which is a highly selective KCNQ current channel blocker (Brown & Passmore, 2009); furthermore, application of XE 991 in the absence of serotonin blocked an outward current that was similar in its voltage dependence to the one induced by serotonin. It has been shown that KCNQ channels require membrane PIP_2_ in order to be active (Brown *et al.* 2007); in our experiments, the PIP_2_ synthesis inhibitor wortmannin prevented the effects of serotonin on LTSIs; this suggests that serotonin may increase the number of active KCNQ channels through an increase in the synthesis of PIP_2_. The observation that wortmannin *per se* depolarised LTSIs indicates that a significant basal level of PIP_2_, sufficient to activate a certain fraction of KCNQ channels, is present in LTSI even in the absence of exogenous serotonin. Activation of 5HT_2C_ receptors stimulates Gq/11, similarly to M1 receptor activation; the latter, however, inhibits, rather than increases, KNCQ conductance through PIP_2_ depletion. Nevertheless, recent results suggest that other Gq_/11_-coupled receptors (such as bradykinin) can actually stimulate PIP_2_ synthesis, possibly by calcium-mediated activation of neuronal calcium sensor-1 and consequent activation of PI(4)-K (Brown *et al.* 2007; Loew, 2007). Further work will be required to ascertain if serotonin-mediated increase in KCNQ channels depends on similar molecular mechanisms. Another possibility is that in LTSIs the effects of 5-HT_2C_ receptor activation may depend on other G proteins such as Gi3 and G13, as found in other cells (Cussac *et al.* 2002).

The inhibition of LTSIs is in stark contrast with the depolarising action of serotonin on two other classes of striatal neurons: the GABAergic fast spiking interneurons and the cholinergic interneurons (Blomeley & Bracci, 2005, 2009). Dopamine also has excitatory actions on fast spiking interneurons and LTSIs (Bracci *et al.* 2002; Centonze *et al.* 2002), while exerting inhibitory effects on cholinergic interneurons (Maurice *et al.* 2004).

The majority of these experiments were carried out in BAC mice that allow easy identification of LTSIs (Partridge *et al.* 2009). In these mice GFP expression is limited, in the striatum, to NPY-expressing interneurons, with no overlap with neurons expressing choline acetyltransferase or parvalbumin (Partridge *et al.* 2009). We selected striatal neurons based on their GFP expression, but only classed them as LTSIs if they displayed the distinctive membrane properties of these neurons (Tepper *et al.* 2010). The effects of serotonin on BAC mice LTSIs were identical to those observed in wild-type rat, showing that the hyperpolarising responses to serotonin were a genuine property of LTSIs and not an artefact due to insertion effects within the BAC promoter construct.

In current-clamp experiments, serotonin was able to either silence spontaneously active LTSIs or dramatically reduce their firing frequency. These results, when integrated with those of previous studies from our and other groups, depict a rather comprehensive picture of the profound changes induced by serotonin on the striatal networks; in the presence of serotonin, glutamatergic inputs to projection neurons will be attenuated (Mathur *et al.* 2011), while fast spiking and cholinergic interneurons will become much more excitable and LTSIs will be strongly inhibited.

The inhibition of LTSIs will result in decreased release of at least four identified neurotransmitters expressed by these cells. In addition to GABA, which inhibits projection neurons (Koos & Tepper, 1999), NPY, somatostatin and nitric oxide are also released by LTSIs. Somatostatin was shown to modulate the membrane properties of projection neurons and to selectively inhibit GABAergic communication between such neurons (Lopez-Huerta *et al.* 2008); there is evidence that NPY facilitates dopamine release (Adewale *et al.* 2007). The effects of NO are more complex and involve corticostriatal communication as well as cholinergic interneurons (Picconi *et al.* 2011; West & Tseng, 2011). Striatal projection neurons have extremely high levels of soluble guanylyl cyclases, the main NO receptors (West & Tseng, 2011) and is involved in both long-term and short-term plasticity (Picconi *et al.* 2011; West & Tseng, 2011).

The multifaceted nature of these effects means that, while we are now in a position to identify most of the cellular and synaptic modifications caused by serotonin release in the striatum, unravelling the overall effect of this neuromodulator on the dynamics of the striatal networks will require large-scale numerical simulations based on realistic synaptic architectures.

## Abbreviations

GFP: green fluorescent protein
ISI: inter-spike interval
LTSI: low-threshold spike interneuron
NPY: neuropeptide Y
